# Hyperhemolysis Syndrome in a Patient with Sickle Cell Disease: A Case Report

**DOI:** 10.5811/cpcem.2020.12.50349

**Published:** 2021-01-26

**Authors:** Joshua A. Kalter, Ranju Gupta, Marna Rayl Greenberg, Andrew J. Miller, Jamie Allen

**Affiliations:** * USF Morsani College of Medicine, Lehigh Valley Health Network, Department of Emergency and Hospital Medicine, Allentown, Pennsylvania; † USF Morsani College of Medicine, Lehigh Valley Health Network, Division of Hematology Oncology, Allentown, Pennsylvania

**Keywords:** Hyperhemolysis, Sickle Cell Disease, disseminated intravascular coagulation

## Abstract

**Introduction:**

Hyperhemolysis syndrome (HHS) is a rare complication of repeat blood transfusions in sickle cell disease (SCD). This can occur acutely or have a delayed presentation and often goes unrecognized in the emergency department (ED) due to its rapid progression and similarity to acute chest syndrome and other common complications of SCD.

**Case Report:**

We present a case of a 20-year-old male with SCD who presented to the ED with pain and tenderness in his lower extremities one day after discharge for a crisis. Unbeknownst to the ED team, during his admission he had received a blood transfusion. On presentation he was noted to have hyperkalemia, hyperbilirubinemia, anemia, and uncontrolled pain, and was admitted for sickle cell pain crisis. Over the next 36 hours, his hemoglobin dropped precipitously from 8.9 grams per deciliter (g/dL) to 4.2 g/dL (reference range: 11.5–14.5 g/dL), reticulocyte count from 11.7 % to 3.8% (0.4–2.2%), and platelets from 318,000 per cubic centimeter (K/cm^3^) to 65 K/cm^3^ (140–350 K/cm^3^). He also developed a fever, hypoxia, transaminitis, a deteriorating mental status, and severe lactic acidosis. Hematology was consulted and he was treated with methylprednisolone, intravenous immunoglobulin, two units of antigen-matched red blood cells, fresh frozen plasma, and cryoprecipitate. He was transferred to an outside hospital for exchange transfusion and remained hospitalized for 26 days with acute liver failure, bone marrow necrosis, and a fever of unknown origin.

**Conclusion:**

Because of the untoward outcomes associated with delay in HHS diagnosis and the need for early initiation of steroids, it is important for emergency providers to screen patients with hemoglobinopathies for recent transfusion at ED presentation. Asking the simple question about when a patient’s last transfusion occurred can lead an emergency physician to include HHS in the differential and work-up of patients with sickle cell disease complications.

## INTRODUCTION

Sickle cell disease (SCD) is a common genetic hemoglobinopathy that has a wide variety of clinical manifestations due to the propensity of deoxygenated hemoglobin to polymerize.^1^,^2^ Sickle hemoglobin (HbS) is one of the variants in this hemoglobinopathy that can lead to hemolytic anemia and vaso-occlusion causing ischemic organ damage, pain crises, stroke, infection, and organ failure.^1^,^2^ Acute vaso-occlusive episodes are generally treated with hydration, antibiotics, pain relief, and, when indicated, red blood cell transfusions.^1^–^4^ Multiple transfusions increase the risk of alloimmunization whereby host antibodies recognize foreign surface antigens on transfused red blood cells and cause hemolysis.^3^,^4^

Hyperhemolysis syndrome (HHS), is a rare hemolytic transfusion reaction characterized by a lower hemoglobin (Hb) than pre-transfusion, fever and pain, decreased reticulocyte count, hyperbilirubinemia, raised lactate dehydrogenase, and hemoglobinuria generally occurring within two weeks of last transfusion.^3^,^4^ The cause of the precipitous drop in Hb remains a debate. King et al proposed the bystander hemolysis mechanism in 1997 followed in 2004 by Petz and Garratty who described any immune hemolysis of cells that are negative for the antigen against which the relevant antibody is directed.^5^,^6^ The other theory, also first described by Petz et al, suggested a transfusion-induced reticulocytopenia and suppression of erythropoiesis.^7^ Complement-mediated lysis and macrophage activation may be a possible mechanism: All these processes are immune-mediated.

The treatment for HHS typically involves immunosuppression with steroids (namely methylprednisolone) as well as intravenous immunoglobulin (IVIG). Hyperhemolysis syndrome is generally divided into an acute and a delayed form based upon its development within seven days vs after seven days of transfusion, respectively. Patients presenting with acute chest syndrome (ACS), aplastic crises, or vaso-occlusive symptoms of SCD may also experience hemoglobinuria, pain, fever, and anemia, making the appropriate diagnosis challenging.^8^,^9^ Rapid diagnosis of HHS is essential as delay in diagnosis can lead to death.^10^

## CASE REPORT

A 20-year-old male with a history of SCD with multiple priapism attacks, ACS, functional asplenia, and sleep apnea presented to the ED with pain and tenderness bilaterally in his lower posterior legs and hips. He had been discharged one day prior from a stay in the hospital for a sickle cell crisis, involving priapism, fever, and generalized pain. Although it was not noted in his intake history, he had received partial exchange transfusion of two units of packed red blood cells (PRBC) four days prior. At the time of arrival, the patient denied any chest pain, shortness of breath, nausea, vomiting, abdominal pain, or genitourinary or gastrointestinal dysfunction including priapism. He reported one episode of a fever at home. Upon examination, there was tenderness to the patient’s bilateral thighs and scleral icterus. Labs revealed a Hb of 8.9 grams per deciliter (g/dL) (reference range: 11.5–14.5 g/dL), white blood count (WBC) of 16.7 thousand per centimeter cubed (K/cm^3^) (4.0–10.0 K/cm^3^), a reticulocyte count of 11.7 (0.4–2.2%), potassium of 5.4 millimoles per liter (mmol)/L) (3.5–5.1 mmol/L), bilirubin of 14.5 milligrams (mg)/dL (0.2–1.0 mg/dL). The patient was admitted for a sickle cell crisis and treated with hydration and analgesics. Of note, historically, the patient’s bilirubin had previously been 6.8 mg/dL (0.2–1.0mg/dL).

The next day, he developed fever (102.6° Fahrenheit), tachycardia, hypoxia, and diminishing mental status. Labs revealed Hb of 6.0 g/dL (11.5–14.5 g/dL), a decrease in platelets from 318 to 154K/cm^3^ (140–350 K/cm^3^), and an increasing WBC count of 17.9K/cm^3^ (4.0–10.0 K/cm^3^). Hematology/oncology was consulted, and the patient was transferred to the intensive care unit out of concern for a delayed hemolytic transfusion reaction. Labs drawn later that night showed a hemoglobin level of 4.2 g/dL (11.5–14.5 g/dL), a reticulocyte count of 3.8 (0.4–2.2%), a platelet count of 65 K/cm^3^ (140–350 K/cm^3^), and schistocytes on peripheral blood smear. The direct antiglobulin test and antibody screen were negative. A disseminated intravascular coagulation (DIC) panel had been ordered due to worsening condition, which showed fibrinogen of less than166 mg/dL (180–500mg/dL) and D-dimer greater than 20 micrograms per milliliter (ug/mL) (less than 0.50ug/ml), prothrombin time of 28.9 seconds (s) (12.0–14.6s), international normalized ratio of 2.8, and partial thromboplastin time of 74.8s (21.6–35.6s).

**CPC-EM Capsule**

What do we already know about this clinical entity?

Hyperhemolysis syndrome (HHS) is a rare complication of repeat blood transfusions in sickle cell disease (SCD).

What makes this presentation of disease reportable?

Lack of information about a prior transfusion in a young male with SCD who presented with declining mental status and fever led to delayed diagnosis of HHS.

What is the major learning point?

Given the outcomes associated with delay in diagnosis and the need for early initiation of steroids, patients with hemoglobinopathies must be screened for recent transfusion.

How might this improve emergency medicine practice?

Asking a patient when their last transfusion occurred can lead the provider to include HHS in the differential and work-up of patients with SCD complications.

Due to continued rapid deterioration and concern for HHS he was started on methylprednisolone and IVIG. Additionally, he was given two units fresh frozen plasma, 10 units cryoprecipitate (due to possible DIC), and 1 unit of PRBC. He was given IVIG (a dose of 0.5 mg/kg) due to rising levels of plasma creatinine and concern for renal injury. The patient needed increasing oxygen by nasal cannula. The differential included HHS, thrombotic thrombocytopenic purpura (TTP) and ACS. Urgent plasmapheresis for possible TTP was attempted but was stopped due to equipment failure. The patient was transferred to an outside hospital for exchange transfusion, where he remained hospitalized with multiorgan failure and persistent fever. Extensive autoimmune and infectious disease workup was done and found to be negative. Despite severe anemia he was not transfused due to the diagnosis of HHS, and he eventually improved after 40 days.

## DISCUSSION

Part of the diagnostic challenge of identifying HHS in SCD is that symptoms are largely similar to those of the general vaso-occlusive crises that patients with SCD experience.^3^–^4^,^8^–^9^ The common symptoms of hemoglobinuria, fever, pain, jaundice and the complications of ACS and liver damage were seen in our patient, and do not differ greatly from his presenting symptoms for previous hospitalizations and are not uncommon for a patient with an underlying disease of chronic hemolysis.^8^,^9^ While decreased reticulocyte count and nadir hemoglobin – lower than presenting values, hyperbilirubinemia, and lactic acidosis are important diagnostic components of hyperhemolysis syndrome,^3^,^4^ they were not seen until the day after admission and coincided with a dramatic decline in the patient’s condition. Additionally, his declining mental status, fever, renal impairment, thrombocytopenia, and microangiopathic hemolytic anemia were initially thought to be from an infectious etiology or TTP.^11^ Although uncommon, TTP has been reported in patients with SCD.^12^,^13^

The key for an emergency physician to successfully make this diagnosis is timing. Per Shah et al, 86.9% of adults and 93.2% of children experience three or less vaso-occlusive crises each year, and moderate-to- severe vaco-occlusive crises often require blood transfusion and hospital admission for several days or longer.^14^ Patients with acute or delayed HHS present within 7–14 days of a blood transfusion. Therefore, an emergency physician should have a high index of suspicion for HHS when a sickle cell patient presents with severe anemia within two weeks of a transfusion. While it is a rare event, with hyperhemolysis reportedly complicating only 3% of transfusions given to patients with SCD,^15^ asking about recent blood transfusions is paramount when trying to differentiate HHS from ACS and these questions should be included when emergency physicians care for patients with SCD. Even though transfusions may not have been indicated in their prior care, for those who have had a recent transfusion, communicating with the admission team and hemoncologist the possibility of HHS allows potentially for earlier, more definitive care and the case evolves in that direction. Our case highlights the importance of screening for recent transfusions in patients with hemoglobinopathies. The subtle differences between HHS and vaso-occlusive crisis in SCD were not readily identifiable until the patient’s condition had markedly declined.

## CONCLUSION

Earlier identification of recent transfusions allows for the appropriate treatment (steroids) for HHS to be initiated. While HHS is rare, it is important for emergency clinicians to screen for prior transfusions in patients with SCD to consider HHS as a diagnosis in these cases.
